# The Relationship of Respiratory Rate-Oxygenation (ROX) and Modified ROX Index With High-Flow Nasal Cannula Oxygen Therapy in COVID-19 Patients: An Observational Pilot Study

**DOI:** 10.7759/cureus.32900

**Published:** 2022-12-24

**Authors:** Habib Md R Karim, Abhishek Bharadwaj, Omer M Mujahid, Manas P Borthakur, Chinmaya K Panda, Jitendra V Kalbande

**Affiliations:** 1 Anesthesiology, Critical Care, and Pain Medicine, All India Institute of Medical Sciences, Raipur, IND; 2 Cardiac Anesthesia and Critical Care, All India Institute of Medical Sciences, New Delhi, IND; 3 Anesthesiology and Critical Care, Sarathi Multi-speciality Hospitals, Nalbari, IND

**Keywords:** prediction, treatment failure, tracheal intubation, non-invasive ventilation, high flow nasal oxygen, artificial respiratory support

## Abstract

Background and aim

Respiratory Rate-Oxygenation (ROX) and modified ROX (mROX) indexes have been proposed to detect early high-flow nasal cannula (HFNC) therapy failure. We evaluated the utility and relationship of ROX and mROX indexes in COVID-19 patients started on HFNC oxygen therapy.

Methods

This pilot study collected data from adult COVID-19 patients requiring HFNC oxygenation from 29 Jan - 29 Jun 2021. The patients were divided into two cohorts based on HFNC therapy success. ROX and mROX were compared using statistical diagnostic testing, including receiver operating characteristics and area under the curve (AUC) using online Epitools (https://epitools.ausvet.com.au/) and MedCalc software (MedCalc Software Ltd, Ostend, Belgium, https://www.medcalc.org/); p<0.05 was considered significant.

Results

Twenty-seven patients fulfilled the inclusion criteria; 48.15% of therapy failed. The cohort's *mean *±* standard deviation* age was 53.93 ± 10.67 years; 74.1% were male. The accuracy of predicting failure for mean ROX versus mROX at baseline and six-hour values was 59.81 versus 70.68 and 67.42 versus 74.88, respectively (all p>0.05). The AUC for ROX and mROX at baseline and at six hours were statistically indifferent. Only an mROX of 4.05 (mean value) and 3.34 (Youden’s J cut-off) had a sensitivity plus specificity at 156% and 163%, respectively.

Conclusion

Both ROX and mROX at baseline and six hours had fair-to-good accuracies and AUC; the differences were insignificant. Both ROX and mROX had better accuracies at six hours. However, only mROX < 4.05 at six hours fulfilled the sensitivity plus specificity criteria to be a clinically valuable screener.

## Introduction

Severe acute respiratory syndrome following the Coronavirus disease of 2019 (COVID-19) has been a global pandemic for nearly two and a half years. These patients are often hypoxemic and need oxygen supplementation. The high-flow nasal cannula (HFNC) oxygen (O2) therapy is newer yet commonly used in critical care during acute hypoxemic respiratory failure (AHRF) arising from postoperative pulmonary complication, pulmonary edema, heart failure, and even in mild hypercapnia respiratory failure. The World Health Organization and other scientific societies list HFNC O2 among the options for ventilatory support. A recent systemic review indicates that HFNC O2 therapy may reduce the need for invasive ventilation and escalation of therapy compared to conventional oxygen therapy in COVID-19 patients with AHRF [1]. Also, in their recent guidelines on managing critically ill adults with COVID-19, the Surviving Sepsis Campaign recommends using HFNC over non-invasive positive pressure ventilation (NIPPV) [2].

However, HFNC oxygenation might delay identifying the need for intubation that would have become necessary. This delay is associated with a poorer prognosis [3]. Roca et al. have formulated and subsequently validated the diagnostic accuracy of an index termed Respiraratory Rate-Oxygenation (ROX) and defined as the ratio of peripheral oxyhemoglobin saturation (SpO2) and the fraction of inspired O2 (FiO2) to respiratory rate (RR) for determining HFNC failure. They demonstrated that the ROX index could assist the clinician in deciding whether or not to intubate patients on HFNC O2 therapy for AHRF [4].

It has been seen that the respiratory rate of patients with COVID-19-related AHRF is sometimes lower than those with other AHRF due to other common causes like pneumonia, and acute pulmonary edema, which can be attributed to the severe level of hypoxia associated with it. As the ROX index uses respiratory rate as the denominator, the ROX index threshold for predicting HFNC O2 therapy failure may be different in the case of COVID-19-associated AHRF. Further, in some patients with COVID-19, the phenomenon of "Happy Hypoxia," or more correctly, "Silent Hypoxia," has been seen [5]. Such patients exhibit shallow oxygen levels (demonstrated by pulse oximetry values) but do not demonstrate dyspnoea, irritability, or other signs of hypoxia. Furthermore, while SpO2/FiO2 has a good correlation with a partial pressure of O2 (PaO2) and FiO2 with low concentrations of supplemental oxygen [6], whether this relationship stands well in patients receiving FiO2 of 100% is not well established. Nevertheless, the correlation between SpO2/FiO2 with PaO2/FiO2 and the fall of SpO2 with PaO2 is not linear [7, 8].

As SpO2 is also used as one of the parameters for calculating the ROX index, the accuracy of the ROX index thresholds in predicting HFNC failure in COVID-19 patients might have to be validated again. A relevant modification of the said index has been proposed by Karim and Esquinas, termed the "Modified ROX (mROX) index” [9]. The mROX has proposed using PaO2 in place of SpO2 to calculate the index. The present prospective, observational pilot study was aimed to evaluate the relationship of ROX and mROX index for predicting failure of HFNC O2 therapy in COVID-19 patients with AHRF.

The article's preprint was submitted to the Research Square platform on May 23, 2022, which is available from https://assets.researchsquare.com/files/rs-1680854/v1/ececdb62-5bd8-4648-a922-ecf5d12c423b.pdf?c=1653317976, last accessed Dec 24, 2022. 

## Materials and methods

The present observational pilot study was conducted in a tertiary care teaching and research institute in India. The data collection was done from 29 Jan 2021 to 29 Jun 2021. The research protocol was reviewed by the research cell, approved by the institutional ethical committee, and subsequently registered in the clinical trial registry of India prospectively (CTRI/2021/01/030431). The study was initially approved to enrol up to 65 years of age; however, it was later amended to include up to 70 years age groups. The study follows the Declaration of Helsinki and the Good Clinical Practice Guideline for biomedical research. The study result is reported per the Strengthening the Reporting of Observational Studies in Epidemiology guideline.

Patients admitted with COVID-19 infection requiring intensive care management and on HFNC oxygen therapy for oxygen supplementation were screened for enrolment. The inclusion criteria were patients aged 18-70 years on HFNC oxygen therapy with continuous SpO2 and RR monitoring. Patients with known pre-existing respiratory diseases like chronic obstructive pulmonary disease (COPD), bronchial asthma, idiopathic pulmonary fibrosis, active cardiac diseases, and chronic renal failure with pulmonary edema were excluded. The sample size was calculated using an online calculator: https://www.crutzen.net/n.htm [10]. We hypothesized a 10% probability of finding a difference between the ROX and mROX and considered a 95% confidence, resulting in a sample size of 29 patients.

Data were collected by observing the patients' parameters from the multiparameter monitor, files, and reports. The investigating team did not intervene in any phase of the treatment of the patients. All patients were on continuous peripheral oxyhemoglobin saturation (SpO2) monitoring; arterial blood gas (ABG) analysis was often done to assess the progress of the artificial respiratory support and escalation-de-escalation of support. All patients put on HFNC O2 therapy were approached for recruitment, and only consenting patients were enrolled. Over and above demographic and history of illness-related data, respiratory rate (RR), SpO2, and the fraction of inspired O2 (FiO2) data were collected every six hours till the patient was either weaned from HFNC or therapy was converted to NIPPV (Bi-level) or invasive mechanical ventilation (IMV). RR, SpO2, and FiO2 data were collected whenever the treating team collected an ABG sample. Further, when the treating team decided to intubate or escalate from HFNC, considering it a failure, RR, SpO2, FiO2, and ABG data were also collected at that time point. ROX and mROX index values were calculated from these data. HFNC failure was defined as the need for either NIV (Bi-level) or IMV, as decided by the attending physician. Usually, decreased level of consciousness (Glasgow Coma Score <12), cardiac arrest/arrhythmias, and severe hemodynamic instability requiring norepinephrine (>0.1μg/kg/min) or persisting or worsening respiratory conditions are defined as at least two of the following criteria: failure to achieve adequate oxygenation (PaO2 <60 mmHg or SpO2 <90% despite HFNC flow ≥60L/min and FIO2 of 100%), respiratory acidosis (PaCO2 >55 mmHg or with pH <7.25), RR >30/min or inability to clear secretions were the factors considered for determining the failure. However, a strict protocol for adherence was absent, but the investigating team noted these criteria-related data at the failure time point. The disease severity was assessed by acute physiology, chronic health evaluation II (APACHE II) score at 24 hours, and organ failure by sequential organ failure assessment (SOFA).

Continuous variables are reported as mean ± standard deviation** **(SD) or median [interquartile range (IQR)] when appropriate. The differences between the two groups were analyzed by Student’s t-test or Mann-Whitney U test. Categorical variables are reported as numbers and percentages and analyzed using Fisher's exact or Chi-square test. INSTAT software (Graphpad Prism Software Inc., La Jolla, USA) was used for this analysis. The receiver operating characteristic (ROC) curves, area under the ROC curves (AUC), and Youden’s J point analysis were done online using Epitools-Epidemiological Calculators (https://epitools.ausvet.com.au/). Diagnostic test evaluation statistical tests for accuracies were done using MedCalc (https://www.medcalc.org/) free statistical calculator online. A two-tailed p-value < 0.05 was considered significant.

## Results

A total of 32 patients were screened for eligibility; one was excluded per the age criteria, and four patients' HFNC oxygen therapy was deemed ineffective within a few minutes of admission. Data from the rest of the 27 patients were included for final analysis. Three-fourths of the data were from male patients. The mean ± SD** **age of the patients was 53.93 ± 10.67** **years. The clinicodemographic data are presented in Table 1.

**Table 1:** Clinico-demographic data and their comparison among success and failure subgroups of the cohort. CKD- chronic kidney disease, AKI- acute kidney injury, OSA- obstructive sleep apnoea, COPD- chronic obstructive pulmonary disease. Note: self or family-reported weight and heights were also included when the measurement was unavailable.

Parameters	All (N=27)	Success (N=14)	Failure (N=13)	Two-tailed p
Age in Years	53.93 ± 10.67	53.93 ± 11.32	53.93 ± 10.39	0.999
Male	20 (74.1)	10 (71.4)	10 (76.9)	0.948
Female	7 (25.9)	4 (28.6)	3 (23.1)	0.948
Weight in kilogram	65.63 ± 9.89	68.36 ± 9.25	62.69 ± 10.06	0.330
Height in cm	168.44 ± 5.79	168.71 ± 6.49	168.15 ± 5.16	0.969
Body mass index	23.14 ± 3.46	24.13 ± 3.91	22.08 ± 2.64	0.303
Diabetes Mellitus	5 (18.52)	2 (14.29)	3 (23.1)	0.948
Hypertension	9 (33.3)	4 (28.57)	5 (38.46)	
CKD/AKI on CKD	1 (3.7)	0	1 (7.69)	
OSA/ COPD/ Asthma	3 (11.11)	1 (7.14)	2 (15.38)	
Other Comorbidities	4 (14.8)	2 (14.29)	2 (15.38)	

Thirteen (48.15%) of the 27 patients' HFNC therapy failed, mostly within 6-24 hours (range 6-96 hours). In the univariate analysis, SOFA, blood urea nitrogen level at admission, ROX at six hours, and mROX at admission and six hours significantly differed among the success and failure cases (Table 2).

**Table 2:** Comparison of the clinico-laboratorial and monitoring data of the cohorts. APACHE: Acute Physiology and Chronic Health Evaluation, SpO2: peripheral oxyhemoglobin saturation, PaO2: partial pressure of arterial Oxygen, PCO2: partial pressure of arterial Carbon-di-oxide, CXR: chest X-ray, ROX: Respiratory rate-oxygenation index, mROX: modified Respiratory rate-oxygenation index, SOFA: sequential organ failure assessment, FiO2: fraction of inspired O2.

Parameters	All (N=27)	Success (N=14)	Failure (N=13)	Two-tailed P-value
APACHE-II	8.81 ± 2.38	8.28 ± 2.33	9.38 ± 2.39	0.2373
SOFA	3.51 ± 1.55	2.78 ± 0.80	4.30 ± 1.79	0.007
Glasgow Coma Scale	15 (15-15)	15 (15-15)	15 (15-15)	---
Mean Blood Pressure	94.4 ± 10.97	96.92 ± 11.80	91.69 ± 9.72	0.219
Respiratory Rate (per min)	27.29 ± 4.17	26.85 ± 3.95	27.76 ± 4.51	0.58
SpO_2_ in percentage	91.18 ± 4.82	92.5 ± 3.79	89.76 ± 5.54	0.151
PaO_2 _in mmHg	75.53 ± 31.51	83.71 ± 36.80	66.72 ± 22.83	0.16
PCO_2_ in mmHg	34.01 ± 4.97	34.03 ± 4.50	33.98 ± 5.61	0.97
Blood Urea Nitrogen	20.43 ± 11.11	16 ± 6.58	25.20 ± 13.16	0.03
FiO_2_ at admission	0.75 ± 0.19	0.70 ± 0.21	0.82 ± 0.16	0.10
FiO_2 _at six hours	0.84 ± 0.16	0.81 ± 0.16	0.88 ± 0.14	0.254
Quadrants involved in CXR	3.03 ± 0.85	2.78 ± 0.97	3.30 ± 0.63	0.11
ROX on admission	4.87 ± 1.67	5.42 ± 1.73	4.29 ± 1.44	0.07
mROX on admission	3.99 ± 1.95	4.78 ± 2.11	3.15 ± 1.38	0.02
ROX at six hours	4.50 ± 1.41	5.05 ± 1.60	3.92 ± 0.90	0.033
mROX at six hours	4.05 ± 2.30	5.07 ± 2.71	2.95 ± 0.98	0.01
ROX on admission versus at six hours (success)				0.563
ROX on admission versus at six hours (failure)				0.441
mROX on admission versus at six hours (success)				0.752
mROX on admission versus at six hours (failure)				0.683

**Figure 1:**
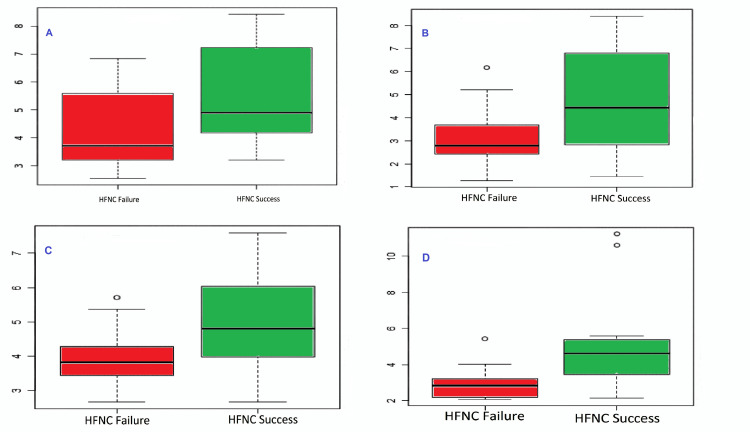
Box and plot representation of ROX at baseline (A), mROX at the baseline (B), ROX at six hours (C), and mROX at six hours (D). ROX: Respiratory rate-oxygenation index, mROX: modified Respiratory rate-oxygenation index, HFNC: high-flow nasal cannula

The mean values of the ROX and mROX at the baseline and six hours were higher for success cases than failure; the difference was significant except for ROX at baseline (Figure 1).

The sensitivity, specificity, accuracy, predictive values, and likelihood ratio analysis showed slightly better values for mROX at six hours. At six hours, the mean mROX value was 4.05, with the highest sensitivity of 87.5%, a positive likelihood ratio of 2.37, and a sensitivity plus specificity of 156%. However, the differences were insignificant statistically (Table 3).

**Table 3:** Diagnostic statistics for assessing accuracies for the indices at the mean values. ROX: Respiratory rate-oxygenation index, mROX: modified Respiratory rate-oxygenation index. Note- *mark indicates that the values were specific for high-flow nasal cannula failure prevalence of 48.15%, as found in our study.

Statistic	Value (95% Confidence Interval)			
	ROX at baseline [mean 4.78]	mROX at baseline [mean 3.98]	ROX at 6 hours [mean 4.5]	mROX at 6 hours [mean 4.05]
Sensitivity (%)	63.64 (30.79 - 89.07)	75.00 (42.81 - 94.51)	72.73 (39.03 - 93.98)	87.50 (47.35 - 99.68)
Specificity (%)	56.25 (29.88 - 80.25)	66.67 (38.38 - 88.18)	62.50 (35.43 - 84.80)	63.16 (38.36 - 83.71)
Positive Likelihood Ratio	1.45 (0.71 - 2.97)	2.25 (1.02 - 4.94)	1.94 (0.94 - 4.02)	2.37 (1.25 - 4.52)
Negative Likelihood Ratio	0.65 (0.26 - 1.58)	0.38 (0.13 - 1.06)	0.44 (0.15 - 1.23)	0.20 (0.03 - 1.28)
Positive Predictive Value (*)	57.46 (39.84 - 73.37)	67.63 (48.75 - 82.1)	64.30 (46.49 - 78.87)	68.80 (53.66 - 80.77)
Negative Predictive Value (*)	62.49 (40.54 - 80.27)	74.17 (50.29 - 89.07)	71.16 (46.66 - 87.44)	84.47 (45.73 - 97.23)
Accuracy (*)	59.81 (39.32 - 78.06)	70.68 (50.14 - 86.47)	67.42 (46.80 - 84.06)	74.88 (54.58 - 89.44)
Sensitivity + Specificity	119.85% (1.198)	141.6% (1.416)	135.2% (1.352)	150.66% (1.566)

**Figure 2:**
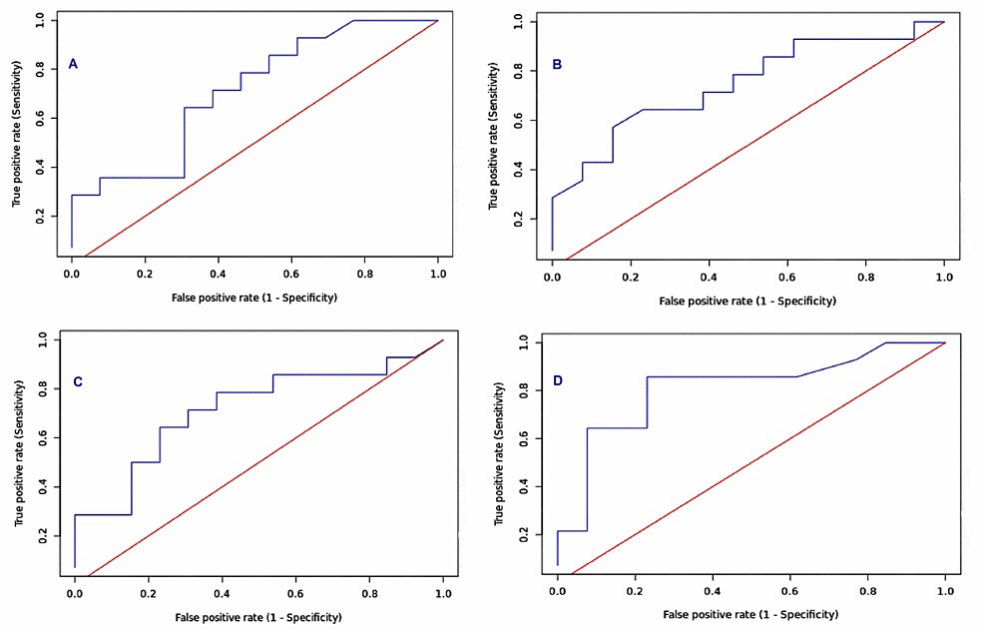
ROC curves for ROX at baseline (A), mROX at baseline (B), ROX at six hours (C), and mROX at six hours (D). ROC: Receiver operating characteristic, ROX: Respiratory rate-oxygenation index, mROX: modified Respiratory rate-oxygenation index.

Similarly, the area under the ROC curve was also highest for mROX at six hours, i.e., 0.81 (95% CI 0.636 - 0.985), while the ROX at six hours had an AUC of 0.717 (95%CI 0.512 - 0.92). The same for the ROX baseline was 0.712 (95% CI 0.512 - 0.911), while the mROX baseline was 0.747 (95% CI 0.558 - 0.937). The ROC curves for ROX and mROX at baseline and six hours are presented in Figure 2. 

The Youden's J cut-off point for ROX and mROX at baseline was 4.59, 4.1, and at six hours was 4.47 and 3.34, respectively. The sensitivity and specificity at Youden's J cut-off points for the ROX and mROX and their efficacy at p=0.01 are presented in Table 4. While the Youden’s J cut-off point value for mROX at baseline (i.e., 4.1) had the highest specificity of 84.6%, the Youden’s J cut-off point value for mROX at six hours (i.e., 3.34) had the highest sensitivity of 85.7%, and sensitivity plus specificity of 162.6%.

**Table 4:** The sensitivity and specificity at Youden's J cut-off points for the ROX and mROX and their efficacy at p=0.01. ROX: Respiratory rate-oxygenation index, mROX: modified Respiratory rate-oxygenation index.

Index/time points	Parameters	Cut-point	Sensitivity	Specificity
ROX at baseline	Youden's J	4.59	0.643	0.692
	Efficacy at P = 0.01	7.23	0.286	1
mROX at baseline	Youden's J	4.1	0.571	0.846
	Efficacy at P = 0.01	6.8	0.286	1
ROX at six hours	Youden's J	4.47	0.643	0.769
	Efficacy at P = 0.01	6.04	0.286	1
mROX at six hours	Youden's J	3.34	0.857	0.769
	Efficacy at P = 0.01	5.57	0.214	1

## Discussion

In the present observational pilot study, the mROX showed slightly better but statistically insignificant predictive accuracy for HFNC failure than the ROX index of respective time points. These findings are in line with Roca et al.’s analysis, where they also found that the diagnostic accuracies of ROX index with SpO2/FiO2 and PaO2/FiO2 (mROX index) were statistically indifferent at two, six, and 12-hours [11]. Unfortunately, the efficacy of both ROX and mROX was only fair to good. None of the indexes at baseline or six-hour could achieve a sensitivity and specificity near 90%. The maximum sensitivity was achieved by mROX at six hours (i.e., 87.55%) at a cut-off value of 4.05, but the specificity was only 63%. While a screening tool does not require high specificity, high sensitivity is desirable to detect most true positive cases. In that context, ROX at six hours and mROX at baseline and six hours appear to have the potential, as they all have a sensitivity of >70%. Nonetheless, for a screening test to be clinically helpful, both sensitivity and specificity are essential, and a criterion based on sensitivity + specificity of 1.5 and above is considered [12]. The only screening test that qualified our pilot cohort's criteria was mROX at six hours. The sensitivity and specificity of the ROX index in our study were like the other contemporary studies and meta-analyses [13, 14].

The present study hypothesis was based on the clinicopathological features of COVID-19 pneumonia and acute respiratory distress syndrome (ARDS). As respiratory rate and oxygenation are altered in COVID-19 AHRF patients compared to non-COVID-19 AHRF patients, we assumed that the ROX and mROX index may have altered efficacy in predicting HFNC O2 therapy in COVID-19 patients. The probable explanation for failing mROX to find better predictors in the present study might be the need for higher FiO2 during HFNC therapy. These patients are mostly moderate-to-severely ill and usually do not require 100% FiO2. At the relatively lower FiO2, SpO2/FiO2, and PaO2/FiO2 correlate well even in COVID-19 patients [15]. As the need for FiO2 increases toward 100% in patients receiving HFNC O2 therapy, the chances of HFNC O2 therapy failure also increase.

In their landmark study, Roca et al. reported the ROX index at 12 hours as having the best prediction accuracy (area under ROC 0.74 [95% confidence interval, 0.64-0.84]) with the best cut-off point for ROX index estimated to be 4.88 [3]. Vega et al. validated the utility of the ROX index as a predictor of HFNC failure for COVID-19 patients [16] and reported similar results to Roca et al. The 12-hour ROX index was the best predictor of intubation with an AUC of 0.7916 [CI 95% 0.6905-0.8927], and the best threshold was 5.99 [Specificity 96%, Sensitivity 62%]. Even the multivariate analysis to determine the predictive factors for HFNC failure by Lun et al. found the ROX index at 12 hours as one of the significant entities [17]. However, the time point for the best prediction is different. Ferrer et al. found the ROX index a good predictor in their observational study [18]. They reported the ROX index at 24 hours as the best predictor of success (AUC 0.826, 95%CI 0.593-1.00) with a cut-off point of 5.35 (Sensitivity 0.91, Specificity 0.79, positive predictive value [PPV] 0.92, negative predictive value [NPP] 0.79). On the other hand, Suliman et al. [19], enrolling COVID-19 patients, found that ROX on day one was a significant predictor of intubation through regression analysis.

In their systematic review and meta-analysis, Prakash et al. assessed the ROX index as a predictor of HFNC failure in COVID-19 patients with AHRF; eight retrospective studies (n=1301 patients) were considered for analysis [20]. The meta-analysis yielded a summary area under the curve (sAUC) of 0.81 (95% CI, 0.77-0.84) with a sensitivity of 0.70 (95% CI, 0.59-0.80) and specificity of 0.79 (95% CI, 0.67-0.88) of ROX index for predicting HNFC failure. The positive and negative likelihood ratio was 3.0 (95% CI, 2.2-5.3) and 0.37 (95% CI, 0.28-0.50), respectively, and was strongly associated with good predictive accuracy (diagnostic odds ratio 9, 95% CI, 5-16). Similarly, the systematic review and meta-analysis by Zhou et al. for ROX index as a predictor of HFNC outcome in pneumonia patients with AHRF found good predictive performance for successful HFNC weaning in patients with an area under ROC of 0.81 (95% CI 0.77-0.84), a pooled sensitivity and specificity of 0.71 (95% CI 0.64-0.78) and 0.78 (95% CI 0.70-0.84), respectively [21]. The analyses also suggested that the ROX index was a reliable predictor of HFNC success in patients with COVID-19 pneumonia. The AUC analysis of our cohort for both ROX and mROX index at admission and six hours was also in line with these studies.

Although most studies using ROX have stressed HFNC failure, Suliman et al. studied the ROX and mROX for predicting the intubation of COVID-19 patients [19]. At the time of intubation, the ROX and mROX median (min-max) values were 3.88 (3.33-6.09) and 5 (3.14-5.52), respectively. In contrast to Suliman et al.’s study [19], 11 out of 13 failure patients were initially tried on Bilevel pressure support non-invasive ventilation before intubation and invasive mechanical ventilation. Nevertheless, all but one patient ultimately required IVM. The mROX and ROX values at six hours and 12 hours in our failure cohort were within the range of the study by Suliman et al. [19], which required intubation, indicating the efficiency of the indexes for not only predicting HFNC failure but also predicting intubation. Rochwerg et al. performed a systematic review and meta-analysis to evaluate the safety and efficacy of HFNC in patients with acute hypoxemic respiratory failure. Nine randomized controlled trials were included (n=2093 patients) and analyzed. They reported that HFNC usage reduced the need for invasive mechanical ventilation compared to conventional oxygen therapy (RR 0.85 95% CI 0.74-0.99 with low certainty) [22].

Further, the use of HFNC reduced the need for escalation of therapy, although it did not affect ICU and hospital length of stay. Notably, delaying intubation leads to increased mortality. In this context, using either the ROX or mROX indexes to predict failure and intubation might be an excellent clinical practice despite needing more sensitivity, specificity, and other limitations [23].

The other intriguing observational finding is the ROX or mROX values trend. Persistent lower values and decreasing trends over 12 hours or 24 hours were the characteristics of the cohort that failed the HFNC. The findings also corroborate with the data presented by Suliman et al. [19] where the cohort requiring intubation also showed a decreasing ROX value over 72 hours.

In the present study, mROX and ROX were almost similar, except for a marginally higher predictive accuracy of mROX at six hours. A recent study taking the readings obtained four hourlies over 24 hours and using the average value also showed that mROX might be better than ROX [24]. However, our study is limited because it was an observational study conducted in a single center and a pilot study. The decision to convert artificial respiratory support from HFNC to NIV (bilevel positive airway pressure - BiPAP) or IMV was at the treating physician's discretion, and no written or strict protocol was followed. We intended to identify the better screener at the earliest and therefore stressed primarily within six hours of the HFNC application. Although the patients were followed until they were either discharged or in heavenly abode, we needed more data to analyze at delayed time points. Sedation is another factor that impacts the tolerance and respiratory rate during non-invasive artificial respiratory support. It bears importance in the context that the sedation requirements of COVID-19 patients are very high and variable [25]. We did not monitor the sedation levels and drugs used for the same, which might have had a minor impact on our results. 

## Conclusions

Both ROX and mROX at baseline and six hours had fair-to-good sensitivity, specificity, positive and negative predictive values, and area under the ROC curve; the differences were statistically insignificant. The accuracies of the indices were better at six hours than the baseline. Although both the indices can be used, only mROX at six hours, with a mean value of 4.05 and a cut-off value of 3.34 had a sensitivity plus specificity at 1.56 and 1.63 (i.e., 156% and 163%), respectively, can be considered a clinically valuable screener. A falling trend or failure to improve the index values over time and progressively increasing need for FiO2 is a vital observation to predict progression towards HFNC oxygenation failure.
